# Supplementary figures and images for: Addition of L-cysteine to the N- or C-terminus of the all-D-enantiomer [D(KLAKLAK)2] increases antimicrobial activities against multidrug-resistant Pseudomonas aeruginosa, Acinetobacter baumannii and Escherichia coli
Source: PeerJ. 2020 Nov 30;8:e10176. doi: 10.7717/peerj.10176 (PMC7713595; doi:10.7717/peerj.10176)

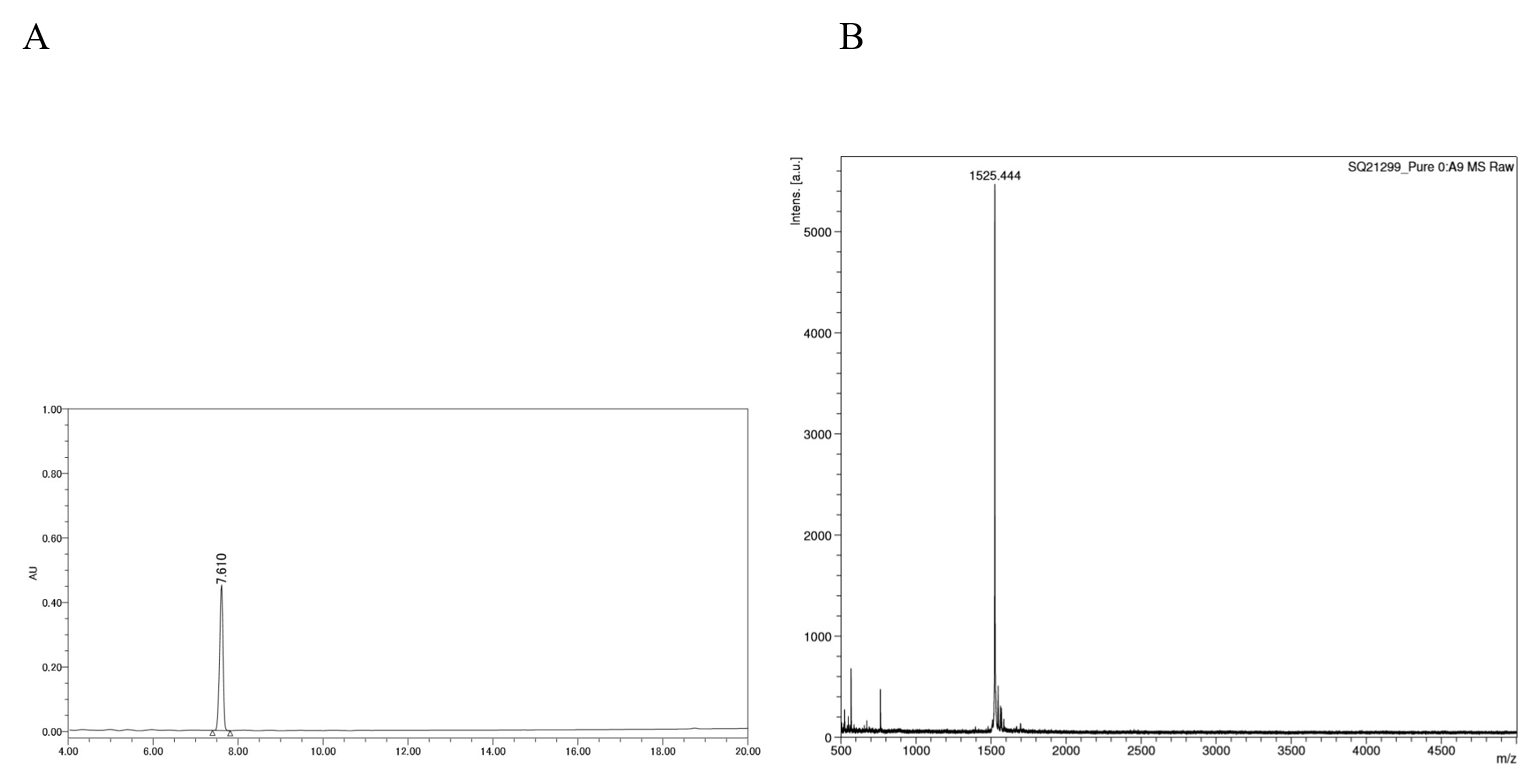

Supplement: Figure S1 — (A) Chromatogram of DP using reversed phase HPLC. DP was loaded onto a SunFire C18 column and eluted with a linear acetonitrile gradient (10–60%) in 20 min. (B) Mass spectra of DP. [file peerj-08-10176-s001.png]

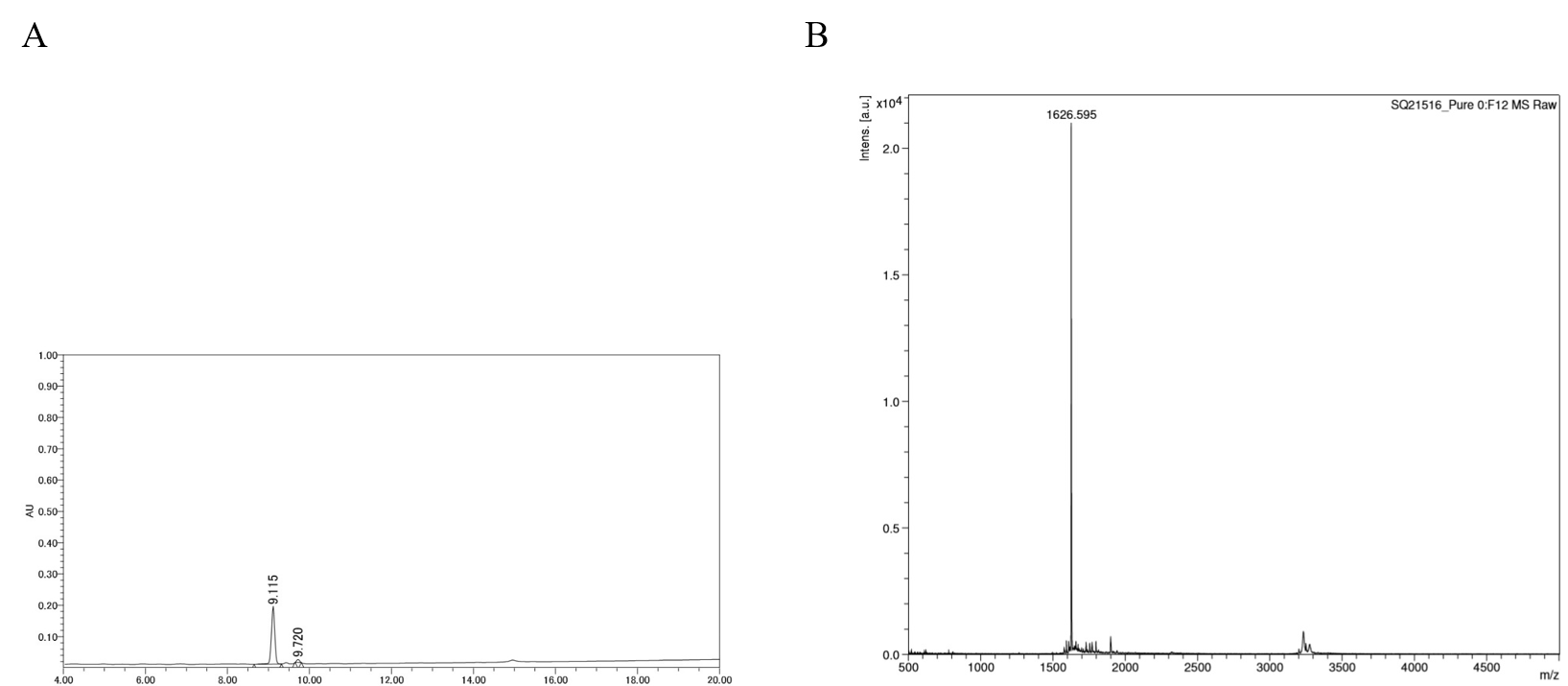

Supplement: Figure S2 — (A) Chromatogram of C-DP using reversed phase HPLC. C-DP was loaded onto a SunFire C18 column and eluted with a linear acetonitrile gradient (10–60%) in 20 min. (B) Mass spectra of C-DP. [file peerj-08-10176-s002.png]

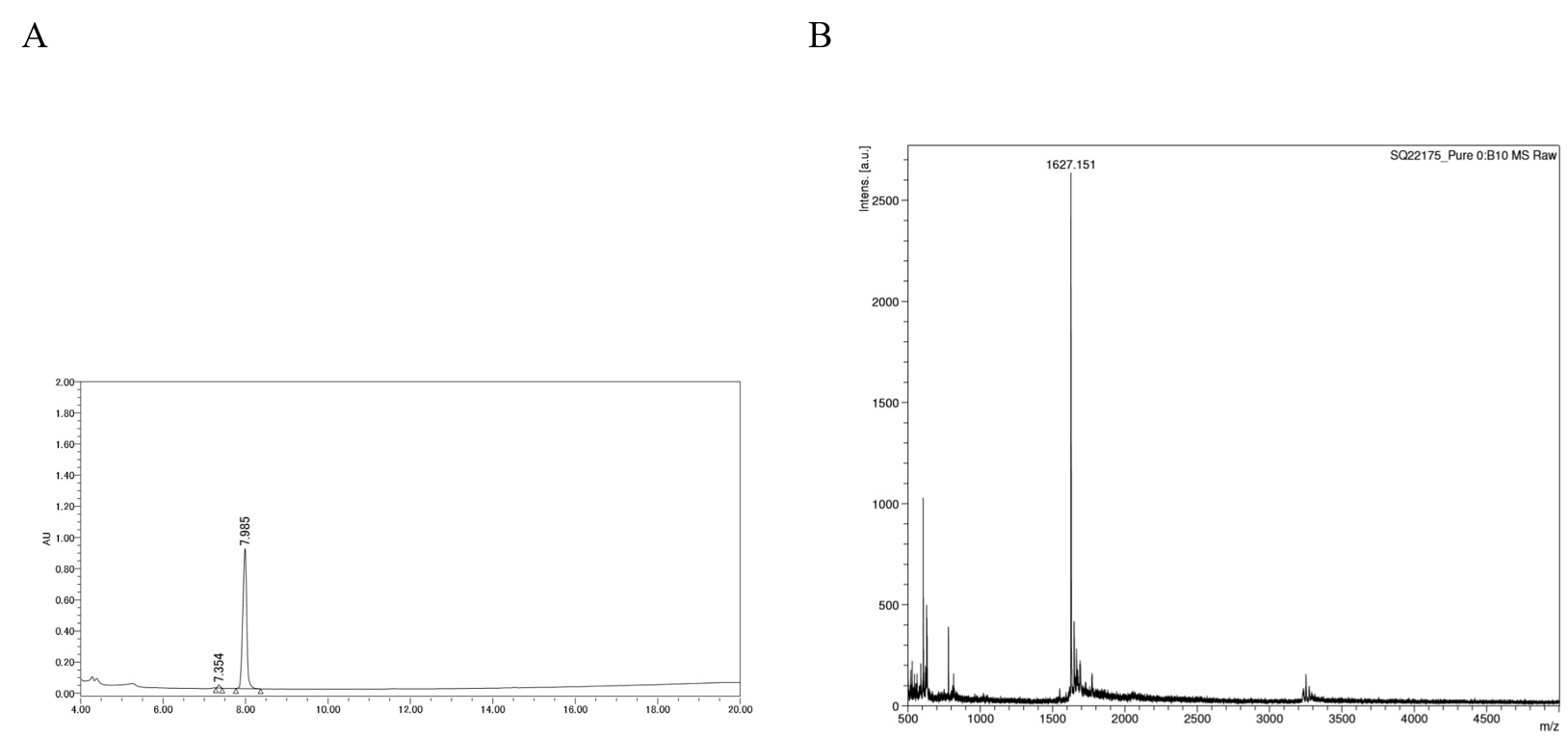

Supplement: Figure S3 — (A) Chromatogram of DP-C using reversed phase HPLC. DP-C was loaded onto a SunFire C18 column and eluted with a linear acetonitrile gradient (0–100%) in 20 min. (B) Mass spectra of DP-C. [file peerj-08-10176-s003.png]

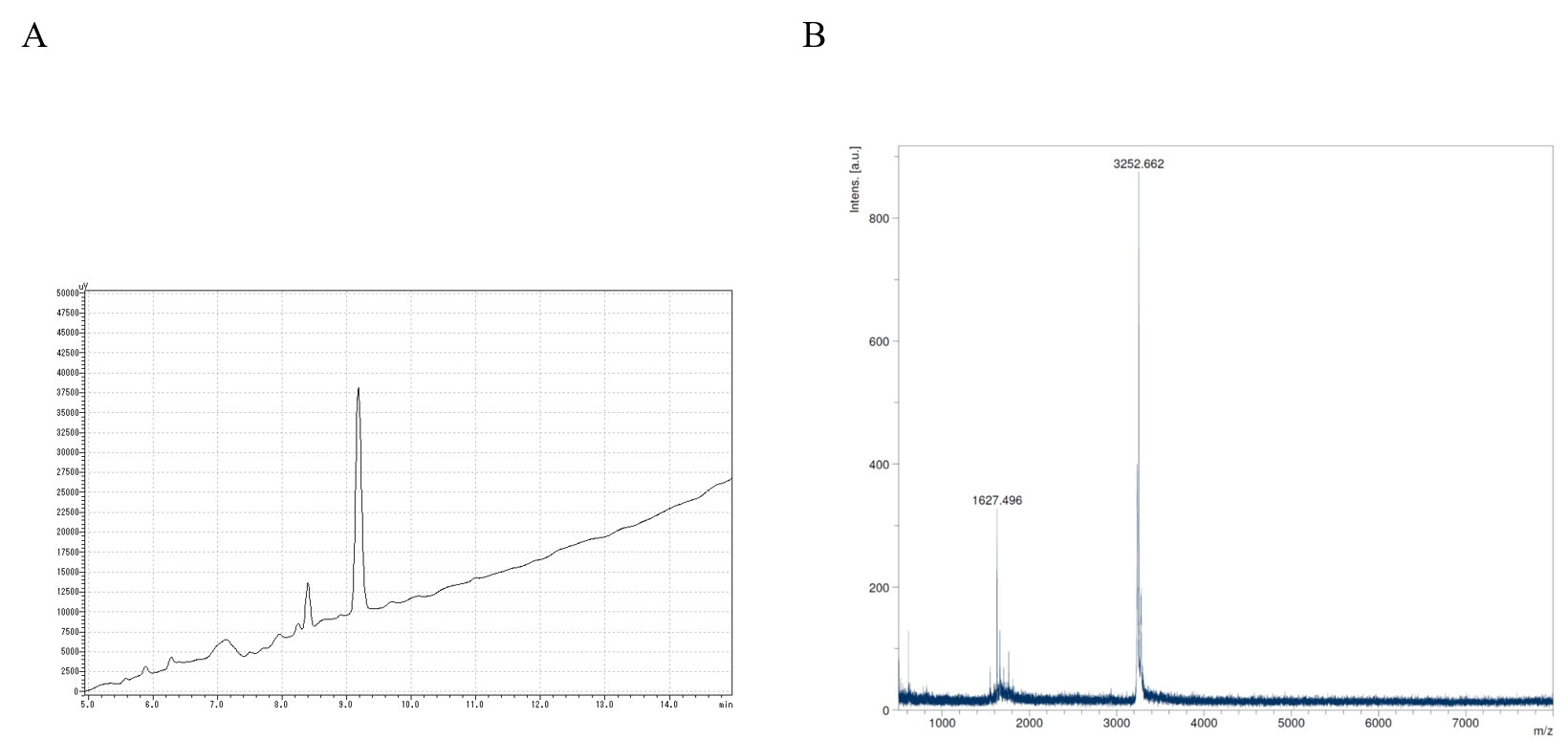

Supplement: Figure S4 — (A) Chromatogram of DP-C dimer using reversed phase HPLC. DP-C dimer was loaded onto a COSMOSIL 5C18-AR-300 column and eluted with a linear acetonitrile gradient (20–50%) in 20 min. (B) Mass spectra of DP-C dimer. [file peerj-08-10176-s004.png]

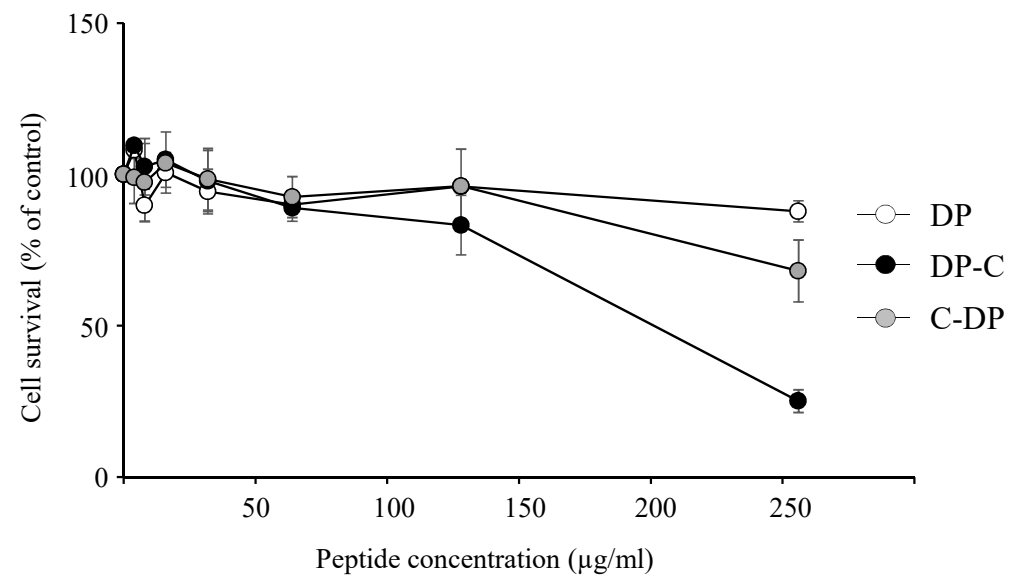

Supplement: Figure S5 — HepG2 cells were seeded at 3,000 cells/well in 96-well microtiter plates. After incubation for 48 h, DP (0–256 µg/ml), C-DP (0–256 µg/ml) or DP-C (0–256 µg/ml) were added, then the plates incubated for an additional 48 h. Cell viability was determined using a Cell Counting Kit-8, with colorimetric changes assessed at OD450 nm with a microplate reader. [file peerj-08-10176-s005.pdf]

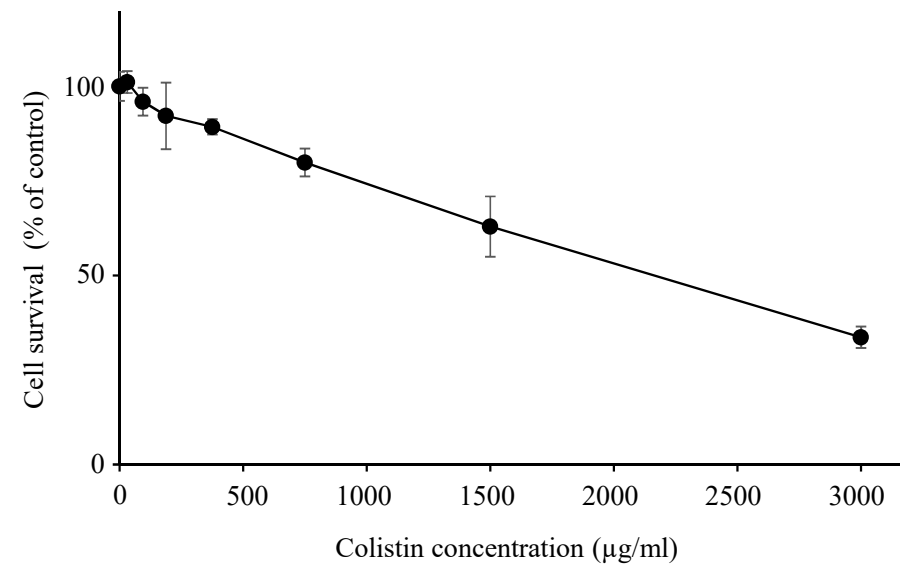

Supplement: Figure S6 — HepG2 cells were seeded at 3,000 cells/well in 96-well microtiter plates. After incubation for 48 h, colistin (0–3,000 µg/ml) were added, then the plates incubated for an additional 48 h. Cell viability was determined using a Cell Counting Kit-8, with colorimetric changes assessed at OD450 nm with a microplate reader. [file peerj-08-10176-s006.pdf]
